# Association of Early Adulthood Hypertension and Blood Pressure Change With Late-Life Neuroimaging Biomarkers

**DOI:** 10.1001/jamanetworkopen.2023.6431

**Published:** 2023-04-03

**Authors:** Kristen M. George, Pauline Maillard, Paola Gilsanz, Evan Fletcher, Rachel L. Peterson, Joseph Fong, Elizabeth Rose Mayeda, Dan M. Mungas, Lisa L. Barnes, M. Maria Glymour, Charles DeCarli, Rachel A. Whitmer

**Affiliations:** 1 Department of Public Health Sciences, University of California Davis School of Medicine, Davis; 2 Department of Neurology, University of California Davis School of Medicine, Sacramento; 3 Division of Research, Kaiser Permanente, Oakland, California; 4 School of Public and Community Health Sciences, University of Montana, Missoula; 5 Department of Epidemiology, Fielding School of Public Health, University of California, Los Angeles, Los Angeles; 6 Rush Alzheimer’s Disease Center, Rush Medical College, Chicago, Illinois; 7 Department of Epidemiology and Biostatistics, University of California, San Francisco, San Francisco

## Abstract

**Importance:**

The association between hypertension developed before midlife and late-life brain health is understudied and, because of the cardioprotective benefits of estrogen before menopause, may differ by sex.

**Objective:**

To assess the association of early adulthood hypertension and blood pressure (BP) change with late-life neuroimaging biomarkers and examine potential sex differences.

**Design, Setting, and Participants:**

This cohort study used data from the Study of Healthy Aging in African Americans (STAR) and Kaiser Healthy Aging and Diverse Life Experiences (KHANDLE) study, which were harmonized longitudinal cohorts of racially and ethnically diverse adults aged 50 years and older from the San Francisco Bay area and Sacramento Valley in California. The STAR was conducted from November 6, 2017, to November 5, 2021, and the KHANDLE study was conducted from April 27, 2017, to June 15, 2021. The current study included 427 participants from the KHANDLE and STAR studies who received health assessments between June 1, 1964, and March 31, 1985. Regional brain volumes and white matter (WM) integrity were measured via magnetic resonance imaging between June 1, 2017, and March 1, 2022.

**Exposures:**

Hypertension status (normotension, transition to hypertension, and hypertension) and BP change (last measure minus first measure) were assessed at 2 multiphasic health checkups (MHCs; 1964-1985) in early adulthood (ages 30-40 years).

**Main Outcomes and Measures:**

Regional brain volumes and WM integrity were measured using 3T magnetic resonance imaging and *z* standardized. General linear models adjusted for potential confounders (demographic characteristics and study [KHANDLE or STAR]) were used to assess the association of hypertension and BP change with neuroimaging biomarkers. Sex interactions were tested.

**Results:**

Among 427 participants, median (SD) ages were 28.9 (7.3) years at the first MHC, 40.3 (9.4) years at the last MHC, and 74.8 (8.0) years at neuroimaging. A total of 263 participants (61.6%) were female and 231 (54.1%) were Black. Overall, 191 participants (44.7%) had normotension, 68 (15.9%) transitioned to hypertension, and 168 (39.3%) had hypertension. Compared with participants who had normotension, those who had hypertension and those who transitioned to hypertension had smaller cerebral volumes (hypertension: β = −0.26 [95% CI, −0.41 to −0.10]; transition to hypertension: β = −0.23 [95% CI, −0.44 to −0.23]), with similar differences in cerebral gray matter volume (hypertension: β = −0.32 [95% CI, −0.52 to −0.13]; transition to hypertension: β = −0.30 [95% CI, −0.56 to −0.05]), frontal cortex volume (hypertension: β = −0.43 [95% CI, −0.63 to −0.23]; transition to hypertension: β = −0.27 [95% CI, −0.53 to 0]), and parietal cortex volume (hypertension: β = −0.22 [95% CI, −0.42 to −0.02]; transition to hypertension: β = −0.29 [95% CI, −0.56 to −0.02]). Participants with hypertension also had smaller hippocampal volume (β = −0.22; 95% CI, −0.42 to −0.02), greater ventricular volumes (lateral ventricle: β = 0.44 [95% CI, 0.25-0.63]; third ventricle: β = 0.20 [95% CI, 0.01-0.39]), larger free water volume (β = 0.35; 95% CI, 0.18-0.52), and lower fractional anisotropy (β = −0.26; 95% CI, −0.45 to −0.08) than those who had normotension. Holding hypertension status constant, a 5-mm Hg increase in systolic BP was associated with smaller temporal cortex volume (β = −0.03; 95% CI, −0.06 to −0.01), while a 5-mm Hg increase in diastolic BP was associated with smaller parietal cortex volume (β = −0.06; 95% CI, −0.10 to −0.02). The negative association of hypertension and BP change with regional brain volumes appeared stronger in men than women for some regions.

**Conclusions and Relevance:**

In this cohort study, early adulthood hypertension and BP change were associated with late-life volumetric and WM differences implicated in neurodegeneration and dementia. Sex differences were observed for some brain regions whereby hypertension and increasing BP appeared more detrimental for men. These findings suggest that prevention and treatment of hypertension in early adulthood is important for late-life brain health, particularly among men.

## Key Points

**Question:**

Are hypertension and blood pressure change in early adulthood associated with late-life brain health, and is there variation by sex?

**Findings:**

In this cohort study of 427 adults 50 years and older, hypertension and increasing blood pressure in early adulthood were associated with lower mean regional brain volumes and poorer white matter integrity in late life. These associations were stronger in men compared with women for some regions.

**Meaning:**

These findings suggest that prevention and treatment of hypertension in early adulthood has important implications for late-life brain health and may be especially important for men.

## Teaser

This cohort study assesses the association of early adulthood hypertension and blood pressure change with late-life neuroimaging biomarkers and examines potential sex differences among adults aged 50 years and older.

## Introduction

Approximately 30% to 50% of the burden of Alzheimer disease and related dementias (ADRDs) is associated with modifiable risk factors,^1^ with hypertension being one of the most common.^2,3^ Midlife hypertension is associated with late-life cognitive decline^4,5,6^ and incident dementia.^7,8,9,10^ Guidelines from the American College of Cardiology/American Heart Association (ACC/AHA)^11,12,13^ propose lower blood pressure (BP) cutoffs for diagnosing hypertension, resulting in an increase in the number of Americans who meet hypertension criteria by 44% to an overall prevalence of 46%. Hypertension prevalence is higher in men (48%) compared with women (43%).^13^ However, after age 65 years, prevalence in women is higher,^13,14^ likely because women lose the cardioprotective benefits of estrogen after menopause.^14,15^

Neuroimaging studies have found an association between midlife hypertension and ADRD pathology,^16,17^ neurodegeneration,^2,18^ and white matter hyperintensities (WMHs).^18,19,20^ In addition, hypertension is believed to be associated with subtle hemodynamic alterations in the brain indicative of early WM degeneration that can be measured via free water (FW) and fractional anisotropy (FA).^21,22,23^ These pathological brain changes often begin decades before dementia onset, suggesting that efforts to prevent and treat hypertension to reduce ADRD burden are needed before midlife.^24^ These efforts are especially important for racial and ethnic minority groups, such as older Asian, Black, and Latino adults in the US, among whom older Black and Latino adults experience a disproportionate risk of dementia.^25^ Older Black adults have an excess risk of hypertension, and all 3 groups (older Asian, Black, and Latino adults) have a low prevalence of hypertension control compared with their White counterparts.^2,25,26^ Older racial and ethnic minority adults are underrepresented in neuroimaging studies, and few studies have evaluated the association of hypertension before midlife with late-life neuroimaging biomarkers.^27,28,29,30^

Using data from the Kaiser Healthy Aging and Diverse Life Experiences (KHANDLE) study and the Study of Healthy Aging in African Americans (STAR), which included harmonized cohorts of older Asian, Black, Latino, and White adults, we assessed the association between hypertension and BP measured at 2 time points in early adulthood (ages 30-40 years) with late-life neuroimaging biomarkers of neurodegeneration and WM integrity. We hypothesized that ACC/AHA-defined hypertension as well as an increase in systolic or diastolic BP over time, regardless of hypertension status, would be associated with lower regional brain volumes, evidence of poor WM integrity, and WMHs. We hypothesized that this association would be stronger in men compared with women because of the cardioprotective benefits of estrogen before menopause.^31^

## Methods

The KHANDLE (conducted from April 27, 2017, to June 15, 2021) and STAR (conducted from November 6, 2017, to November 5, 2021) were harmonized longitudinal cohort studies of long-term Kaiser Permanente Northern California members living in the San Francisco Bay area and Sacramento Valley. The similar study designs and data collection methods of the KHANDLE and STAR allowed for direct comparison across studies and pooling of the 2 cohorts. These studies aimed to understand life-course factors associated with cognitive aging and ADRD. Participants were eligible for the KHANDLE study if they were 65 years or older on January 1, 2017, spoke English or Spanish, and participated in multiphasic health checkups (MHCs) as part of routine care.^32,33,34^ Stratified random sampling by race and ethnicity and educational attainment was used to recruit approximately equal proportions of Asian, Black, Latino, and White participants. Participants eligible for the STAR self-identified as Black or African American, were 50 years or older on January 1, 2018, participated in MHCs, and spoke English.^35^ Stratified random sampling by age and educational attainment was used to recruit approximately equal proportions of Black participants aged 50 to 64 years and 65 years and older. Exclusion criteria for both studies included an electronic medical record diagnosis of dementia or other neurodegenerative disease and the presence of health conditions that would impede participation in study interviews.^35,36^ The current cohort study included 427 participants from the KHANDLE and STAR studies who received health assessments in early adulthood (ages 30-40 years) between June 1, 1964, and March 31, 1985. Both the KHANDLE and STAR studies were approved by the institutional review boards of Kaiser Permanente and the University of California, Davis. All participants provided written informed consent, which included permission to use and publish their data in future analyses. The current study followed the Strengthening the Reporting of Observational Studies in Epidemiology (STROBE) reporting guideline for cohort studies.

The MHCs were offered as part of routine care at Kaiser Permanente Northern California.^35,36^ To be eligible for enrollment, participants in the KHANDLE and STAR studies had to complete at least 1 visit between 1964 and 1973 or 1 visit between 1977 and 1985; however, they could have participated in up to 5 overlapping waves: 1964 to 1973, 1973 to 1977, 1977 to 1985, 1985 to 1992, and 1992 to 1996. (The years overlap due to minor changes in the types of data collected over time.) For these analyses, participants had to have attended at least 2 MHCs 1 or more years apart. To preserve sample size and maximize time between BP measures, data from the first and last available MHC assessment were used to ascertain hypertension status and BP change. Hypertension was defined using AHA/ACC guidelines^13^ as systolic BP of 130 mm Hg or higher, diastolic BP of 80 mm Hg or higher, or self-report of antihypertensive or heart medication use. We identified participants to have normotension (did not meet hypertension criteria at MHCs), transitioning to hypertension (had normotension at the first MHC and hypertension at the last MHC), or having hypertension (met hypertension criteria at the first MHC). Participants who had hypertension at the first MHC but reverted to normotension at the last MHC were considered to have hypertension. Within-person BP change was calculated by subtracting the first MHC BP measurement from the last MHC BP measurement for both systolic and diastolic BP. All BP measurements were taken by trained clinical staff using standard procedures.^37^

A stratified random sample of approximately 25% of KHANDLE and STAR participants were selected for brain imaging using 3T magnetic resonance imaging (MRI; Tim Trio; Siemens) housed in the Imaging Research Center of the University of California Davis Medical Center (eTable 1 in Supplement 1). A total of 591 participants completed neuroimaging between June 1, 2017, and March 1, 2022. Well-established MRI sequences for assessment of neuronal injury were collected, including 2-dimensional fluid-attenuated inversion recovery for WMHs and high angular resolution diffusion tensor imaging. Variables created by the scans included atlas-based regional cortical brain volumes, hippocampal volume, and lobar WM lesion volumes. Diffusion tensor imaging–based measurements of FW and FA, which are sensitive indicators of WM integrity, were assessed using algorithms described elsewhere.^38,39,40,41^

Several covariates were included as potential confounders in the association of early adulthood hypertension status and BP change with late-life neuroimaging biomarkers. Covariates included race and ethnicity (self-reported as Asian, Black, Latino, or White), age at imaging (calculated using date of birth and imaging examination date), age at first MHC (calculated using date of birth and MHC date), study (KHANDLE or STAR), sex (male or female), and educational attainment (high school diploma or less, some college or trade school, college degree, or graduate degree). Race and ethnicity data were relevant to the study due to the well-established differences in prevalence and incidence of hypertension and dementia by race and ethnicity. Sex was derived from self-report or participant medical records and likely reflected a mixture of biological sex and gender identity. We assumed both sex and gender referred to biological sex for this study due to our focus on biological differences. Educational attainment was derived from self-reported highest completed grade or degree at baseline interviews.

### Statistical Analysis

Of 591 participants included in the neuroimaging subsample, 164 were excluded (140 had 1 MHC from 1964-1985, 15 had <1 year elapse between their first and last MHC, and 9 were missing any imaging measurements), resulting in a final analytic sample of 427 participants. Residual values for all brain regions of interest were used in analyses and estimated by regressing each region on total cranial volume, with the exception of FW and FA.^42^ All brain region values were *z* standardized for comparison across regions of interest. The natural log of WMH volume was used to account for the right skew of the data. Inverse probability weights were calculated to account for potential selection bias in the neuroimaging subsample. Weights were calculated by estimating the inverse probability of selection for inclusion into the analytic MRI sample from the full KHANDLE and STAR cohorts based on study, systolic BP at the first MHC, diastolic BP at the first MHC, age at the first MHC, race and ethnicity, sex, educational attainment, and age at KHANDLE or STAR baseline. Weights were stabilized on race and ethnicity, sex, and educational attainment.

We estimated medians and the prevalence of baseline characteristics in the neuroimaging subsample stratified by hypertension status and provided baseline characteristics for the full STAR and KHANDLE cohorts used to create the selection weights. Weighted general linear models were used to assess the association of hypertension status (normotension, transition to hypertension, or hypertension) with each brain region of interest and measurement of WM integrity. We used cubic splines to evaluate nonlinear associations of systolic and diastolic BP with regional brain volumes and found that associations were linear. Thus, weighted general linear models were also used to estimate associations of systolic and diastolic BP change with each brain region of interest and measurement of WM integrity. All models were adjusted for race and ethnicity, study, age at first MHC, time between first and last MHC, age at neuroimaging, sex, and educational attainment. Models assessing systolic and diastolic BP were run separately and also adjusted for hypertension status. The interactions of hypertension status and sex and BP change and sex were tested. We report 95% CIs throughout but tested sex interactions using a significance threshold of 2-tailed *P* < .10 due to sample size. We used β coefficients to interpret the differences in mean values of the outcome per unit increment of the exposure after covariate adjustment.

As a sensitivity analysis, we reran models examining hypertension status and BP change restricted to participants with first and last MHCs that were between 6 and 16 years apart, representing the 25th to 75th percentiles (ie, IQR) for time between first and last MHC. All analyses were conducted using SAS software, version 9.4 (SAS Institute Inc).

## Results

Among 427 participants, median (SD) ages were 28.9 (7.3) years at the first MHC, 40.3 (9.4) years at the last MHC, and 74.8 (8.0) years at neuroimaging (Table 1). A total of 164 participants (38.4%) were male, 263 (61.6%) were female, 69 (16.2%) were Asian, 231 (54.1%) were Black, 65 (15.2%) were Latino, and 61 (14.3%) were White (information on race and ethnicity was missing for 1 participant [0.2%]). Overall, 191 participants (44.7%) had normotension, 68 (15.9%) transitioned to hypertension, and 168 (39.3%) had hypertension.

**Table 1.** Baseline Characteristics of Neuroimaging Subsample

Characteristic	Participants, No. (%)				
	Neuroimaging subsample by hypertension status				Total KHANDLE and STAR cohort (N = 2477)^a^
	Total (n = 427)	Normotension (n = 191)	Transition to hypertension (n = 68)	Hypertension (n = 168)	
Age at neuroimaging, median (SD), y^b^	74.8 (8.0)	73.9 (8.3)	76.8 (7.0)	74.9 (7.9)	NA
Age at wave 1, median (SD), y^c^	72.6 (7.8)	71.7 (8.2)	74.9 (6.8)	72.6 (7.7)	74.1 (8.0)
Sex					
Male	164 (38.4)	48 (25.1)	32 (47.1)	84 (50.0)	802 (32.4)
Female	263 (61.6)	143 (74.9)	36 (52.9)	84 (50.0)	1675 (67.6)
Race and ethnicity					
Asian	69 (16.2)	30 (15.7)	12 (17.6)	27 (16.1)	339 (13.7)
Black	231 (54.1)	109 (57.1)	29 (42.6)	93 (55.4)	1164 (47.0)
Latino	65 (15.2)	28 (14.7)	11 (16.2)	26 (15.5)	261 (10.5)
White	61 (14.3)	24 (12.6)	15 (22.1)	22 (13.1)	291 (11.7)
Missing	1 (0.2)	0	1 (1.5)	0	422 (17.0)
Educational attainment					
High school diploma or less	58 (13.6)	27 (14.1)	11 (16.2)	20 (11.9)	304 (12.3)
Some college or trade school	173 (40.5)	72 (37.7)	26 (38.2)	75 (44.6)	792 (32.0)
College degree	94 (22.0)	43(22.5)	14 (20.6)	37 (22.0)	502 (20.3)
Graduate degree	102 (23.9)	49 (25.7)	17 (25.0)	36 (21.4)	459 (18.5)
Missing	0	0	0	0	420 (17.0)
Age at MHC, median (SD), y					
First visit	28.9 (7.3)	29.0 (7.6)	29.1 (7.3)	28.9 (7.0)	29.1 (7.1)
Last visit	40.3 (9.4)	38.9 (9.4)	40.9 (9.2)	40.3 (9.5)	NA
Systolic BP at MHC, median (SD), mm Hg					
First visit	120.0 (14.2)	112.0 (9.3)	116.0 (9.6)	131.0 (12.4)	120.0 (14.1)
Last visit	117.0 (16.3)	110.0 (9.4)	130.0 (16.4)	124.0 (16.5)	NA
Diastolic BP at MHC, median (SD), mm Hg					
First visit	70.0 (11.1)	68.0 (7.4)	68.0 (9.2)	80.1 (9.9)	70.0 (11.0)
Last visit	71.0 (10.1)	67.0 (6.5)	80.0 (9.3)	74.0 (10.3)	NA

Abbreviations: BP, blood pressure; KHANDLE, Kaiser Healthy Aging and Diverse Life Experiences; MHC, multiphasic health checkup; NA, not applicable; STAR, Study of Healthy Aging in African Americans.

^a^ All participants in the STAR cohort were of Black race. Participants in the KHANDLE cohort were of Asian, Black, Latino, and White race and ethnicity.

^b^ Neuroimaging was performed between April 1, 2017, and March 1, 2022.

^c^ Wave 1 in the KHANDLE study was from April 27, 2017, to December 20, 2018. Wave 1 in the STAR was from November 6, 2017, to March 2, 2020.

Participants who transitioned to hypertension (median [SD] age, 76.8 [7.0] years) between MHCs were approximately 2 years older than those who had hypertension (median [SD] age, 74.9 [7.9] years) and approximately 3 years older than those who had normotension (median [SD] age, 73.9 [8.3] years) (Table 1).Educational attainment was highest among participants with normotension (49 [25.7%] had a graduate degree vs 17 [25.0%] who transitioned to hypertension and 36 [21.4%] who had hypertension). Median BP was highest in the group who had hypertension at the first MHC (systolic BP: median [SD], 131.0 [12.4] vs 116.0 [9.6] among those who transitioned to hypertension and 112.0 [9.3] among those who had normotension; diastolic BP: median [SD], 80.1 [9.9] vs 68.0 [9.2] among those who transitioned to hypertension and 68.0 [7.4] among those who had normotension); however, at the last MHC, BP was highest in the group who transitioned to hypertension (systolic BP: mean [SD], 130.0 [16.4] vs 124.0 [16.5] among those who had hypertension and 110.0 [9.4] among those who had normotension; diastolic BP: mean [SD], 80.0 [9.3] vs 74.0 [10.3] among those who had hypertension and 67.0 [6.5] among those who had normotension). This difference may reflect uptake of antihypertensive medication among participants in the hypertension group. Compared with the full KHANDLE and STAR cohort, participants selected for the MRI sample were slightly younger but had comparable systolic and diastolic BP at their first MHC.

### Hypertension Status

We first evaluated mean differences in the volumes of brain regions of interest by hypertension status using weighted general linear models (Table 2). Participants who transitioned to hypertension or had hypertension in early adulthood had significantly smaller mean cerebral volume (transition to hypertension: β = −0.23 [95% CI, −0.44 to −0.02]; hypertension: β = −0.26 [95% CI, −0.41 to −0.10]), cerebral gray matter volume (transition to hypertension: β = −0.30 [95% CI, −0.56 to −0.05]; hypertension: β = −0.32 [95% CI, −0.52 to −0.13]), frontal cortex volume (transition to hypertension: β = −0.27 [95% CI, −0.53 to 0]; hypertension: β = −0.43 [95% CI, −0.63 to −0.23]), and parietal cortex volume (transition to hypertension: β = −0.29 [95% CI, −0.56 to −0.02]; hypertension: β = −0.22 [95% CI, −0.42 to −0.02]) compared with participants who had normotension at both MHC assessments. Participants with hypertension had significantly smaller mean hippocampal volume (β = −0.22; 95% CI, −0.42 to −0.02) as well as larger mean lateral ventricle volume (β = 0.44; 95% CI, 0.25-0.63) and third ventricle volume (β = 0.20; 95% CI, 0.01-0.39) compared with those with normotension. Hypertension was also associated with larger FW volume (β = 0.35; 95% CI, 0.18-0.52) and lower FA (β = −0.26; 95% CI, −0.45 to −0.08). These findings suggest that transitioning to hypertension and having hypertension in young adulthood are comparable with 1 to 3 years of accelerated brain aging (age: β = − 0.08; 95% CI, −0.09 to −0.06).

**Table 2.** Mean Difference in Volumes of Brain Regions of Interest by Hypertension Status Estimated From General Linear Models^a^

Region of interest	β (95% CI)			*P* value for interaction between hypertension status and sex
	Hypertension status			
	Normotension	Transition to hypertension	Hypertension	
Gray matter volume				
Cerebrum	1 [Reference]	−0.23 (−0.44 to −0.02)	−0.26 (−0.41 to −0.10)	.45
Cerebrum gray	1 [Reference]	−0.30 (−0.56 to −0.05)	−0.32 (−0.52 to −0.13)	.06
Hippocampus	1 [Reference]	−0.14 (−0.40 to 0.12)	−0.22 (−0.42 to −0.02)	.28
Cortex volume				
Frontal	1 [Reference]	−0.27 (−0.53 to 0)	−0.43 (−0.63 to −0.23)	.03
Occipital	1 [Reference]	−0.20 (−0.47 to 0.07)	−0.16 (−0.36 to 0.04)	.59
Temporal	1 [Reference]	−0.08 (−0.34 to 0.19)	−0.07 (−0.27 to 0.13)	.87
Parietal	1 [Reference]	−0.29 (−0.56 to −0.02)	−0.22 (−0.42 to −0.02)	.29
Cerebrospinal fluid volume				
Lateral ventricle	1 [Reference]	0.24 (−0.01 to 0.50)	0.44 (0.25 to 0.63)	.55
Third ventricle	1 [Reference]	0.06 (−0.20 to 0.31)	0.20 (0.01 to 0.39)	.43
White matter integrity				
Free water	1 [Reference]	0.22 (−0.01 to 0.45)	0.35 (0.18 to 0.52)	.40
Fractional anisotropy	1 [Reference]	−0.16 (−0.41 to 0.09)	−0.26 (−0.45 to −0.08)	.09
Log-white matter hyperintensities	1 [Reference]	0.06 (−0.17 to 0.30)	0.17 (−0.01 to 0.35)	.10

^a^ All brain region values were *z* standardized. Models were adjusted for race and ethnicity (Asian, Black, Latino, or White), study (Kaiser Healthy Aging and Diverse Life Experiences [KHANDLE] or Study of Healthy Aging in African Americans [STAR]), age at first multiphasic health checkup (MHC), time between first and last MHC, age at neuroimaging, sex (male or female), and educational attainment (high school diploma or less, some college or trade school, college degree, or graduate degree). Separate interaction models tested for the interaction between hypertension status and sex.

**Figure 1.**
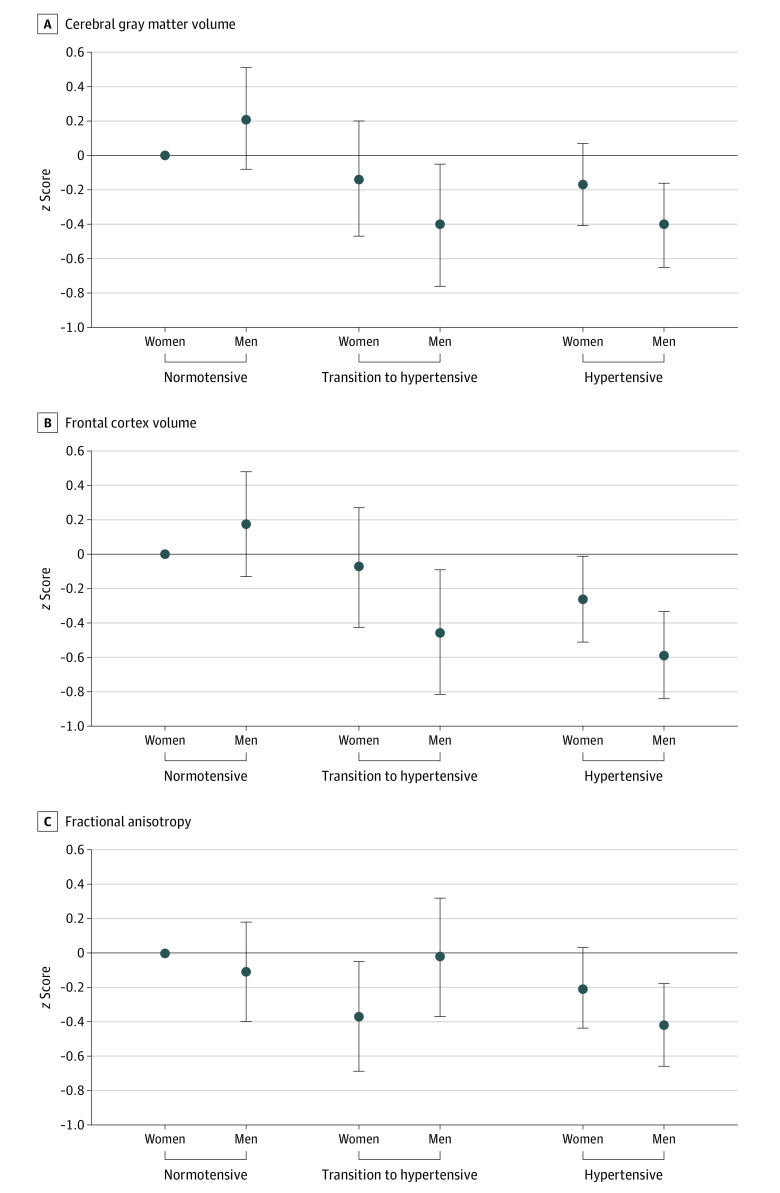
Mean Difference in Cerebral Gray Matter Volume, Frontal Cortex Volume, and Fractional Anisotropy Stratified by Sex and Hypertension Status The reference group was women with normotension. The model was adjusted for race and ethnicity (Asian, Black, Latino, or White), study (Kaiser Healthy Aging and Diverse Life Experiences [KHANDLE] or Study of Healthy Aging in African Americans [STAR]), age at first multiphasic health checkup (MHC), time between first and last MHC, age at neuroimaging, sex (male or female), and educational attainment (high school diploma or less, some college or trade school, college degree, or graduate degree). Whiskers represent 95% CIs. The solid horizontal line at 0 on the y-axis represents the reference line.

Sex differences were observed in the associations of hypertension status with cerebral gray matter and frontal cortex volumes as well as FA (Figure 1). Compared with women with normotension, there were no significant differences in cerebral gray matter volume for men who had normotension (β = 0.21; 95% CI, −0.08 to 0.51), women who transitioned to hypertension (β = −0.14; 95% CI, −0.47 to 0.20), and women who had hypertension (β = −0.17; 95% CI, −0.41 to 0.07); however, cerebral gray matter volume was significantly smaller in men who transitioned to hypertension (β = −0.40; 95% CI, −0.76 to −0.05) and men who had hypertension (β = −0.40; 95% CI, −0.65 to −0.16). Similarly, there was no significant difference in frontal cortex volume observed for men who had normotension (β = 0.18; 95% CI, −0.13 to 0.48) or women who transitioned to hypertension (β = −0.07; 95% CI, −0.42 to 0.27) compared with women who had normotension, but men who transitioned to hypertension (β = −0.46; 95% CI, −0.82 to −0.09), women who had hypertension (β = −0.26; 95% CI, −0.51 to −0.01), and men who had hypertension (β = −0.59; 95% CI, −0.84 to −0.33) had significantly smaller frontal cortex volumes. Differences in FA by sex and hypertension status followed a similar but less distinct pattern. Compared with women who had normotension, FA did not significantly differ for men who had normotension (β = −0.11; 95% CI, −0.40 to 0.18), men who transitioned to hypertension (β = −0.02; 95% CI, −0.37 to 0.32), and women who had hypertension (β = −0.21; 95% CI, −0.44 to 0.03). Women who transitioned to hypertension (β = −0.37; 95% CI, −0.69 to −0.05) and men who had hypertension (β = −0.42; 95% CI, −0.66 to −0.18) had significantly lower FA. Overall, interactions revealed stronger associations between hypertension status and brain volume in men than women.

### Blood Pressure Change

**Figure 2.**
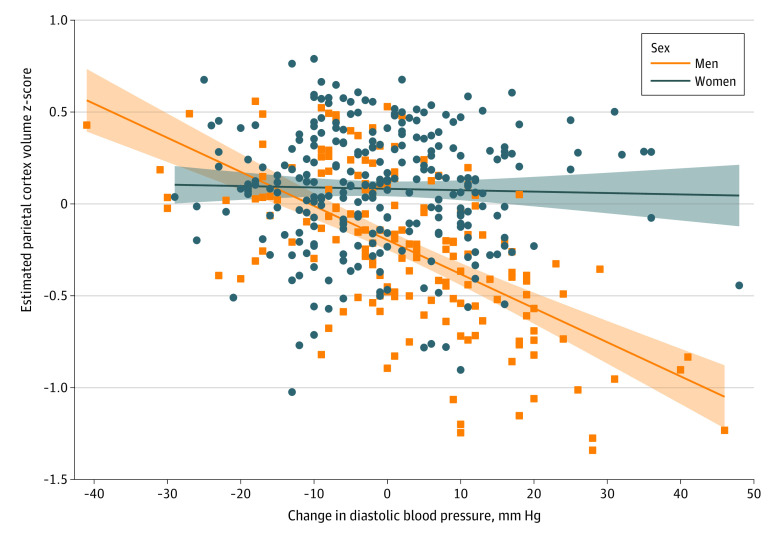
Linear Association Between Parietal Cortex Volume and Change in Diastolic Blood Pressure Between First and Last Multiphasic Health Checkup Stratified by Sex Models were adjusted for race and ethnicity (Asian, Black, Latino, or White), study (Kaiser Healthy Aging and Diverse Life Experiences [KHANDLE] or Study of Healthy Aging in African Americans [STAR]), age at first multiphasic health checkup (MHC), time between first and last MHC, age at neuroimaging, sex (male or female), and educational attainment (high school diploma or less, some college or trade school, college degree, or graduate degree). Blue circles represent parietal cortex volumes for women, and orange squares represent parietal cortex volumes for men. The solid blue line represents the estimated parietal cortex volumes for women across change in diastolic blood pressure, and the blue shading represents 95% CIs. The solid orange line represents the estimated parietal cortex volumes for men across change in diastolic blood pressure, and the orange shading represents 95% CIs.

Change in BP was evaluated by assessing systolic and diastolic BP separately (Table 3). Holding hypertension status constant, a 5-mm Hg increase in systolic BP between the first and last MHC was associated with lower temporal cortex volume (β = −0.03; 95% CI, −0.06 to −0.01). A 5-mm Hg increase in diastolic BP between the first and last MHC was associated with significantly lower parietal cortex volume (β = −0.06; 95% CI, −0.10 to −0.02). There was a sex interaction for the association between diastolic BP and parietal cortex volume whereby the negative association was significantly stronger in men compared with women (Figure 2).

**Table 3.** Linear Association Between Volumes of Brain Regions of Interest and Change in Blood Pressure Between First and Last Multiphasic Health Checkup^a^

Region of interest	β (95% CI)	*P* value for interaction between systolic BP change and sex	β (95% CI)	*P* value for interaction between diastolic BP change and sex
	Systolic BP per 5-mm Hg increase		Diastolic BP per 5-mm Hg increase	
Gray matter volume				
Cerebrum	−0.01 (−0.04 to 0.01)	.11	−0.01 (−0.05 to 0.02)	.83
Cerebrum gray	−0.02 (−0.05 to 0.01)	.60	−0.03 (−0.07 to 0.01)	.23
Hippocampus	−0.01 (−0.04 to 0.02)	.22	0.01 (−0.03 to 0.05)	.57
Cortex volume				
Frontal	−0.01 (−0.04 to 0.01)	.69	0.01 (−0.03 to 0.05)	.54
Occipital	−0.01 (−0.04 to 0.02)	.43	0 (−0.04 to 0.04)	.38
Temporal	−0.03 (−0.06 to −0.01)	.91	−0.03 (−0.07 to 0.01)	.95
Parietal	0 (−0.03 to 0.03)	.90	−0.06 (−0.10 to −0.02)	.01
Cerebrospinal fluid volume				
Lateral ventricle	0.02 (0 to 0.05)	.69	0.02 (−0.03 to 0.05)	.38
Third ventricle	0 (−0.03 to 0.03)	.28	−0.02 (−0.06 to 0.02)	.27
White matter integrity				
Free water	0.01 (−0.01 to 0.04)	.43	0.01 (−0.02 to 0.05)	.65
Fractional anisotropy	−0.01 (−0.04 to 0.02)	.44	0.01 (−0.03 to 0.04)	.77
Log-white matter hyperintensities	0.01 (−0.02 to 0.03)	.47	0.01 (−0.03 to 0.05)	.83

Abbreviation: BP, blood pressure.

^a^ All brain region values were *z* standardized. Models were adjusted for race and ethnicity (Asian, Black, Latino, or White), study (Kaiser Healthy Aging and Diverse Life Experiences [KHANDLE] or Study of Health Aging in African Americans [STAR]), age at first multiphasic health checkup (MHC), time between first and last MHC, age at neuroimaging, sex (male or female), and educational attainment (high school diploma or less, some college or trade school, college degree, or graduate degree). Separate interaction models tested for the interaction between BP change and sex.

### Sensitivity Analysis

Time between first and last MHC differed substantially within the cohorts. We restricted analyses to those with a first and last MHC fell within the IQR of 6 to 16 years. Findings for hypertension status (eTable 2 in Supplement 1) and BP change (eTable 3 in Supplement 1) were largely the same. Some results were attenuated and no longer significant, but all estimates were in the expected direction. Sex interactions were not tested in this restricted sample due to the small sample size.

## Discussion

In this cohort study involving a racially and ethnically diverse cohort of older adults, we found that compared with participants who had normotension in early adulthood, transitioning to hypertension was associated with lower cerebral, cerebral gray matter, frontal cortex, and parietal cortex volumes in late life. Early adulthood hypertension was associated with smaller cerebral, cerebral gray matter, hippocampal, frontal cortex, and parietal cortex volumes as well as larger lateral ventricle, third ventricle, and FW volumes and lower FA. We observed sex differences in the association of early adulthood hypertension status with cerebral gray matter and frontal cortex volumes as well as FA whereby associations appeared stronger in men compared with women. When evaluating BP change, a 5-mm Hg increase in systolic BP was associated with lower temporal cortex volume, and a 5-mm Hg increase in diastolic BP was associated with lower parietal cortex volume and differed by sex.

Similar to our findings, a recent review of approximately 30 studies^16^ found that hypertension, measured primarily in mid and late life, was associated with neurodegeneration in the frontal, parietal, and temporal lobes as well as the hippocampus. Previous work^23,43^ has found an association between elevated BP and evidence of WM degeneration measured via FA, FW, and WMH volume. These studies^23,43^ focused on midlife and late-life hypertension among participants who ranged in age from 45 to 80 years and defined hypertension using older and less conservative BP cutoffs.^16^ Our findings suggested that consideration of the presence of hypertension earlier than midlife, defined using the most recent hypertension diagnostic guidelines,^13^ may be important for identifying individuals at high risk of neurodegenerative brain changes associated with dementia, especially those who would have previously been classified to have prehypertension or normotension under older diagnostic criteria.^11^ Furthermore, our findings suggested that regardless of hypertension status, increases in BP in early adulthood were associated with lower volumes in some brain regions of interest. Findings from Insight 46,^44,45^ a British birth cohort study, suggest there may be a sensitive period between early adulthood and early midlife during which increased BP is particularly detrimental to late-life brain health.

When evaluating sex differences, our study found that hypertension and BP change in both men and women were associated with volumetric differences that have been implicated in cognitive impairment and dementia.^17^ These negative associations were slightly stronger in men for some brain regions of interest. There are several potential explanations for these findings. Sex hormones are believed to have a cardioprotective benefit for women until they reach menopause, after which their risk of cardiovascular disease increases to match that of similarly aged men.^15,31^ This phenomenon may protect women from the detrimental consequences of hypertension for the brain, particularly in early adulthood.^15^ There may also be sex differences in other modifiable ADRD risk factors that play a role in sex differences in neuroimaging biomarkers. Findings from the Behavioral Risk Factor Surveillance System^46^ revealed that the combined population-attributable risk of ADRD was higher in men (35.9%) compared with women (30.1%) when evaluating 8 of the most common ADRD risk factors, including hypertension, which had a population-attributable risk of 9.6% in men vs 8.0% in women. However, with limited sample size in the current study, replication of these findings in a larger cohort is needed. Understanding sex differences in the association between hypertension and neuroimaging biomarkers may help to understand sex differences in ADRD outcomes as well as identify sex-specific sensitive periods during which intervention might be most beneficial.

### Strengths and Limitations

This study has several strengths. Our study is an important contribution to the literature because it reveals the importance of early adulthood hypertension status and BP for late-life brain health. We had the benefit of a racially and ethnically diverse cohort, longitudinal data spanning several decades, and the ability to assess hypertension status and BP at 2 time points.

This study also has limitations. Only a subset of KHANDLE and STAR participants had neuroimaging measurements, limiting the sample size. Furthermore, the KHANDLE and STAR studies were designed to recruit racial and ethnic groups that have been historically underrepresented in research in the US, and findings may not generalize to broader populations of older adults, although our findings are consistent with previous work.^16,23,43^ Due to the limited sample size, we were unable to examine racial and ethnic differences, and significant sex interactions should be interpreted with caution. Our study was also limited due to BP measurements with varying time between the 2 measurements. We restricted inclusion to participants with MHC examinations at least 1 year apart and conducted a sensitivity analysis restricted to participants whose MHCs were between 6 and 16 years apart. Having measurements at equal intervals for all participants would have provided additional power for our estimates. Neuroimaging was only available from 1 time point in late life. Thus, we cannot determine whether the observed differences are evidence of neurodegeneration; we can only state that volumetric differences by hypertension status and BP change were observed. Future work will evaluate whether neuroimaging biomarkers mediate the association of early adulthood hypertension and BP change with late-life cognitive change.

## Conclusions

 The findings of this cohort study involving diverse older adults suggested that early adulthood hypertension and BP change were associated with differences in late-life neuroimaging biomarkers that are implicated in cognitive decline and dementia. This work emphasizes the need to better understand how sex and timing of the onset of hypertension and BP change modify the association between hypertension and neurodegeneration.

## Notes

Supplement 1. **eTable 1.** Detailed Neuroimaging Methods Used in STAR and KHANDLE **eTable 2.** Mean Difference in Brain Region of Interest Volumes (*z* Standardized) by Hypertension Status Estimated From General Linear Models Restricted to Participants With MHC Assessments 6 to 16 Years Apart (IQR) **eTable 3.** Linear Association Between Brain Region of Interest Volumes (*z* Standardized) and Change in Blood Pressure Between First and Last MHC Restricted to Participants With MHC Assessments 6 to 16 Years Apart (IQR)

Supplement 2.
Data Sharing Statement
