# Supplementary material for: Association of Early Adulthood Hypertension and Blood Pressure Change With Late-Life Neuroimaging Biomarkers
Source: JAMA Netw Open. 2023 Apr 3;6(4):e236431. doi: 10.1001/jamanetworkopen.2023.6431 (PMC10071343; doi:10.1001/jamanetworkopen.2023.6431)
Supplement: Supplement 1. — eTable 1. Detailed Neuroimaging Methods Used in STAR and KHANDLE eTable 2. Mean Difference in Brain Region of Interest Volumes (z Standardized) by Hypertension Status Estimated From General Linear Models Restricted to Participants With MHC Assessments 6 to 16 Years Apart (IQR) eTable 3. Linear Association Between Brain Region of Interest Volumes (z Standardized) and Change in Blood Pressure Between First and Last MHC Restricted to Participants With MHC Assessments 6 to 16 Years Apart (IQR) [file jamanetwopen-e236431-s001.pdf]

Supplementary Online Content

George KM, Maillard P, Gilsanz P, et al. Association of early adulthood hypertension and blood pressure change with late-life neuroimaging biomarkers. *JAMA Netw Open*. 2023;6(4):e236431. doi:10.1001/jamanetworkopen.2023.6431

eTable 1. Detailed Neuroimaging Methods Used in STAR and KHANDLE

eTable 2. Mean Difference in Brain Region of Interest Volumes (*z* Standardized) by Hypertension Status Estimated From General Linear Models Restricted to Participants With MHC Assessments 6 to 16 Years Apart (IQR)

eTable 3. Linear Association Between Brain Region of Interest Volumes (*z* Standardized) and Change in Blood Pressure Between First and Last MHC Restricted to Participants With MHC Assessments 6 to 16 Years Apart (IQR)

This supplemental material has been provided by the authors to give readers additional information about their work.

© 2023 George KM et al. *JAMA Network Open*.

eTable 1. Detailed Neuroimaging Methods Used in STAR and KHANDLE

**Image Acquisition**	MRIs were performed on a 3T Siemens TrioTrim (version syngo MR B17). Three sequences were used: a T1-weighted volumetric MP-RAGE (3DT1): repetition time (TR)=2500 ms, echo time (TE)=2.98 ms, inversion time (TI)=1100 ms, 192 slices total, FOV=256 mm, acquisition matrix =256 x 256, slice thickness = 1 mm, a fluid attenuated inversion recovery (FLAIR) sequence: TR=8800 ms, TE=500 ms, TI=2360 ms, 96 slices total, FOV=256 mm, acquisition matrix =256 x 256, slice thickness = 2 mm, and a multi-shell diffusion tensor imaging (DTI) sequence: TR=6000 ms, TE=87 ms, 48 slices total, FOV=256 mm, acquisition matrix = 96 × 96, slice thickness = 2.7 mm with 2.7 mm gap. Diffusion weighted images were generated using 13 gradients directions with gradient diffusion sensitivity of b=500 s/mm2, 21 gradients directions with b=1000 s/mm2, 15 gradients directions with b=2000 s/mm2, and 3 images with b=0 s/mm2.
**Brain MRI Analysis**	All images were transferred to and processed by the University of California Davis Medical Center without knowledge of clinical information. Segmentation and quantification of total cerebral cranial volume (TCV) was determined using a convolutional neural network method.[1] Non-linear co-registration of images to the Desikan-Killiany-Tourville atlas[2] enabled calculation of regional gray matter volumes.[3,4] Segmentation of WMHs utilized a Bayesian approach where the likelihood of WMH was estimated from FLAIR signal characteristics, the prior probability of WMH occurrence was calculated from previous supervised segmentations of independent FLAIR images and additional posterior probability constraints were applied at each image voxel.[5] Hippocampal masks were computed by a multi-atlas hippocampal segmentation algorithm.[3] WMH volumes were log-transformed to normalize population variance. Regional MRI volumes were corrected for head size by calculating the percentage of TCV. FW and FA maps were calculated from DTI using a model that considers two co-existing compartments per voxel: one FW compartment which models isotropic diffusion with a diffusion coefficient of water at body temperature (37 °C) and a second compartment, which accounts for all other molecules.[6,7]
**References**	1. Fletcher E, DeCarli C, Fan AP, Knaack A. Convolutional Neural Net Learning Can Achieve Production-Level Brain Segmentation in Structural Magnetic Resonance Imaging. *Front Neurosci*. 2021;15:683426. doi:10.3389/fnins.2021.683426<br>2. Desikan RS, Segonne F, Fischl B, et al. An automated labeling system for subdividing the human cerebral cortex on MRI scans into gyral based regions of interest. *NeuroImage*. Jul 1 2006;31(3):968-980. doi:DOI 10.1016/j.neuroimage.2006.01.021<br>3. Aljabar P, Heckemann RA, Hammers A, Hajnal JV, Rueckert D. Multi-atlas based segmentation of brain images: atlas selection and its effect on accuracy. *NeuroImage*. Jul 1 2009;46(3):726-38. doi:10.1016/j.neuroimage.2009.02.018<br>4. Tustison NJ, Cook PA, Klein A, et al. Large-scale evaluation of ANTs and FreeSurfer cortical thickness measurements. *NeuroImage*. Oct 1 2014;99:166-79. doi:10.1016/j.neuroimage.2014.05.044<br>5. Maillard P, Hillmer LJ, Lu H, et al. Instrumental Validation of Free Water, Peak-Width of Skeletonized Mean Diffusivity and White Matter Hyperintensities: MarkVCID Neuroimaging kits. *Alzheimers Dement (Amst)*. 2022;In production; doi:10.1002/dad2.12261<br>6. Jenkinson M, Beckmann CF, Behrens TE, Woolrich MW, Smith SM. Fsl. *NeuroImage*. Aug 15 2012;62(2):782-90. doi:10.1016/j.neuroimage.2011.09.015<br>7. Hoy AR, Koay CG, Kecskemeti SR, Alexander AL. Optimization of a free water elimination two-compartment model for diffusion tensor imaging. *NeuroImage*. Dec 2014;103:323-333. doi:10.1016/j.neuroimage.2014.09.053

© 2023 George KM et al. *JAMA Network Open*.

eTable 2. Mean Difference in Brain Region of Interest Volumes (*z* Standardized) by Hypertension Status Estimated From General Linear Models Restricted to Participants With MHC Assessments 6 to 16 Years Apart (IQR)

	Normotensive	Transition to Hypertensive	Hypertensive
	**β (95% CI)**	**β (95% CI)**	**β (95% CI)**
**Gray Matter Volumes**			
Cerebrum	Ref	-0.14 (-0.43, 0.15)	-0.34 (-0.57, -0.11)
Cerebrum Gray	Ref	-0.31 (-0.64, 0.04)	-0.46 (-0.73, -0.19)
Hippocampus	Ref	0.04 (-0.32, 0.40)	-0.21 (-0.49, 0.07)
Frontal Cortex	Ref	-0.17 (-0.51, 0.17)	-0.52 (-0.79, -0.26)
Occipital Cortex	Ref	-0.38 (-0.72, -0.04)	-0.08 (-0.34, 0.19)
Temporal Cortex	Ref	-0.17 (-0.54, 0.19)	-0.22 (-0.51, 0.07)
Parietal Cortex	Ref	-0.22 (-0.57, 0.13)	-0.34 (-0.62, -0.07)
**Cerebrospinal Fluid Volumes**			
Lateral Ventricle	Ref	0.08 (-0.27, 0.42)	0.51 (0.24, 0.78)
Third Ventricle	Ref	-0.10 (-0.47, 0.27)	0.17 (-0.12, 0.46)
**Measures of White Matter Integrity**			
Free Water	Ref	0.15 (-0.16, 0.47)	0.30 (0.06, 0.55)
Fractional Anisotropy	Ref	-0.01 (-0.31, 0.30)	-0.19 (-0.43, 0.04)
Log White Matter Hyperintensities	Ref	-0.01 (-0.34, 0.32)	0.04 (-0.22, 0.30)

Models adjust for race, study, age at first MHC, time between first and last MHC, age at neuroimaging, sex, and education

© 2023 George KM et al. *JAMA Network Open*.

eTable 3. Linear Association Between Brain Region of Interest Volumes (*z* Standardized) and Change in Blood Pressure Between First and Last MHC Restricted to Participants With MHC Assessments 6 to 16 Years Apart (IQR)

	**Systolic Blood Pressure (per 5 mm Hg)**	**Diastolic Blood Pressure (per 5 mm Hg)**
	**β (95% CI)**	**β (95% CI)**
**Gray Matter Volumes**		
Cerebrum	-0.03 (-0.06, 0.01)	0.00 (-0.05, 0.05)
Cerebrum Gray	-0.04 (-0.08. -0.01)	-0.03 (-0.09, 0.02)
Hippocampus	-0.00 (-0.05, 0.04)	0.05 (-0.01, 0.10)
Frontal Cortex	-0.04 (-0.08, -0.00)	0.00 (-0.05, 0.05)
Occipital Cortex	-0.02 (-0.06, 0.01)	0.01 (-0.04, 0.07)
Temporal Cortex	-0.04 (-0.08, 0.00)	-0.03 (-0.09, 0.03)
Parietal Cortex	-0.01 (-0.05, 0.03)	-0.07 (-0.12, -0.01)
**Cerebrospinal Fluid Volumes**		
Lateral Ventricle	0.04 (-0.00, 0.07)	-0.02 (-0.08, 0.03)
Third Ventricle	0.02 (-0.03, 0.06)	-0.06 (-0.12, -0.00)
**Measures of White Matter Integrity**		
Free Water	0.02 (-0.02, 0.05)	-0.00 (-0.05, 0.05)
Fractional Anisotropy	-0.01 (-0.05, 0.02)	0.01 (-0.04, 0.06)
Log White Matter Hyperintensities	0.00 (-0.03, 0.04)	0.00 (-0.05, 0.06)

Models adjust for race, study, age at first MHC, time between first and last MHC, age at neuroimaging, sex, and education

© 2023 George KM et al. *JAMA Network Open*.
