# Supplementary material for: Association of Early Adulthood Hypertension and Blood Pressure Change With Late-Life Neuroimaging Biomarkers
Source: JAMA Netw Open. 2023 Apr 3;6(4):e236431. doi: 10.1001/jamanetworkopen.2023.6431 (PMC10071343; doi:10.1001/jamanetworkopen.2023.6431)
Supplement: Supplement 2. — Data Sharing Statement [file jamanetwopen-e236431-s002.pdf]

Data Sharing Statement

George. Association of Early Adulthood Hypertension and Blood Pressure Change With Late-Life Neuroimaging Biomarkers. *JAMA Netw Open*. Published April 03, 2023. doi:10.1001/jamanetworkopen.2023.6431

Data
Data available: Yes
Data types: Deidentified participant data
How to access data: Those interested in using the data can submit a research proposal at this website: https://sites.google.com/g.ucla.edu/khandle-study-site/home.
When available: With publication

Supporting Documents
Document types: None

Additional Information
Who can access the data: Data will be available to researchers whose proposed use has been approved.
Types of analyses: Data will be available for biomedical research and related analyses (e.g., epidemiological studies).
Mechanisms of data availability: Data will be available after approval of a proposal by principal investigators.
